# Effect of Long-Term Consumption of *Lactobacillus paracasei* SD1 on Reducing Mutans streptococci and Caries Risk: A Randomized Placebo-Controlled Trial

**DOI:** 10.3390/dj3020043

**Published:** 2015-04-01

**Authors:** Rawee Teanpaisan, Supatcharin Piwat, Sukanya Tianviwat, Benchamat Sophatha, Thanyanan Kampoo

**Affiliations:** 1 Common Oral Diseases and Epidemiology Research Center and the Department of Stomatology, Faculty of Dentistry, Prince of Songkla University, Hat-Yai 90112, Thailand; E-Mails: benchamat02@gmail.com (B.S.); kanokporn.k@psu.ac.th (T.K.); 2 Common Oral Diseases and Epidemiology Research Center and the Department of Preventive Dentistry, Faculty of Dentistry, Prince of Songkla University, Hat-Yai 90112, Thailand; E-Mails: supacharin.p@psu.ac.th (S.P.); sukanya.ti@psu.ac.th (S.T.)

**Keywords:** *Lactobacillus paracasei* SD1, mutans streptococci, probiotics, dental caries

## Abstract

Background: A previous study revealed *Lactobacillus paracasei* SD1, a probiotic strain, could reduce mutans streptococci (MS). The aim of this study was to evaluate the long-term effects of *L. paracasei* SD1 on the colonization of MS, and whether caries lesions developed. Methods: After informed consent, 122 children were recruited and randomly assigned to the probiotic or control groups. The probiotic group received milk-powder containing *L. paracasei* SD1 and the control group received standard milk-powder once daily for six months. Salivary MS and lactobacilli were enumerated using differential culture at baseline and at three-month intervals for 12 months. The persistence of *L. paracasei* SD1 was investigated using AP-PCR for DNA-fingerprinting. Oral health was examined at baseline and at the end of the study according to WHO criteria. Results: The long-term consumption could prolong colonization of *L. paracasei* SD1. Significantly reduced MS counts and increased lactobacilli levels were found among children in the probiotic group. There were less caries lesions in the probiotic group at the end of the study. A significant reduction of the development of new caries lesions (4.5 times) was observed in the high caries risk group but not in the low caries risk group. Conclusions: Results demonstrate that the long-term daily ingestion of the human-derived probiotic *L. paracasei* SD1 significantly reduces the number of MS and caries risk in the high caries group.

## 1. Introduction

Dental caries still remains one of the most common diseases worldwide, especially in developing countries. A great effort has been made searching for the means to reduce cariogenic microflora, however, this seems to have been unsuccessful in completely eradicating caries-associated microorganisms. A number of studies have reported the use of probiotic strains for the prevention of oral diseases, including dental caries [1,2].

*Lactobacillus* species are major organisms that have been previously evaluated as potential probiotics for the prevention of dental caries. This is mainly due to their purported inhibitory activity against cariogenic mutans streptococci (MS) [3,4,5], and the fact that they are generally considered safe for oral administration in humans [6]. A number of *in vivo* clinical control trials have been performed and most of those demonstrated positive effects of probiotics on reducing MS [2]. Probiotic strains previously used for oral care are those mainly derived from non-oral origins e.g., the gastrointestinal tract. They might not be ideal strains for the oral environment since this differs somewhat from the gastrointestinal habitat. This has been supported by the findings that such strains usually colonize in the oral cavity for only a short time [7,8,9]. However, most of the information received has been from short-term consumption of probiotics. A long term study is needed to clarify how long the probiotics would persist and whether a cariogenic microbial shift would be induced.

Another point of concern is that most studies observed the number of MS as the outcome, while reports of outcomes of caries lesion development are still lacking. This is impossible to do so unless a long-term study is carried out.

Our previous studies demonstrated that *Lactobacillus paracasei* SD1, an oral human-derived strain, is a good candidate to be a probiotic for oral health. The randomized double blinded studies showed that short-term, four-week consumption of milk powder containing *L. paracasei* SD1 could reduce salivary MS in volunteers. The strain could still be found up to four weeks following cessation of dosing [10,11].

The aims of the present study were to investigate the long-term effect of *L. paracasei* SD1 on the colonization of MS, and the persistence of *L. paracasei* SD1 *in vivo* was also evaluated. Moreover, caries lesion development was monitored.

## 2. Results and Discussion

In recent years, bacteriotherapy in the form of probiotic bacteria with an inhibiting effect on oral pathogens has been a promising concept, thus, probiotic applications for prevention and/or treatment of oral diseases have received increased attention. Short- and long-term intervention in clinical trials have been performed, and most have shown that receiving probiotic strains could decrease the MS levels in either saliva or dental plaque. However, some different results have also been reported. This inconsistency has been explained by the different strains used or differences in study design and/or study population [12,13,14,15,16,17,18].

**Figure 1:**
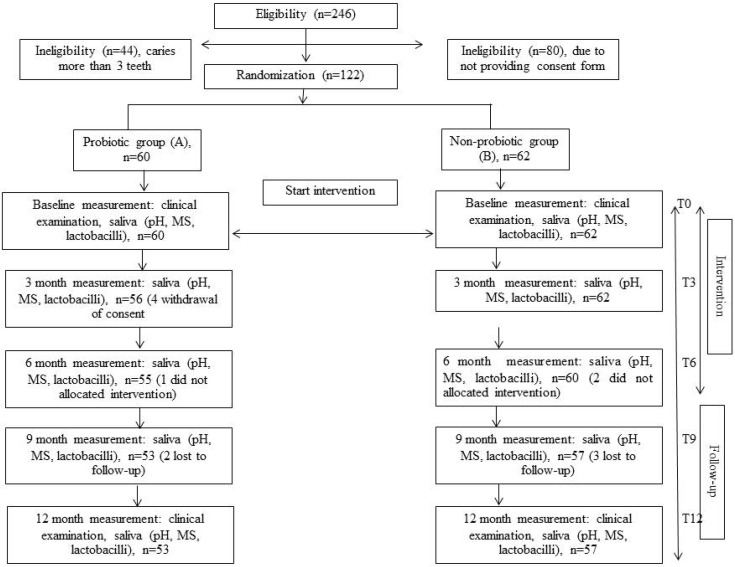
Flow-chart showing the progress of children participating at each time period of the 12-month study.

Most studies on probiotics and dental caries have measured changes in MS counts, but only a few studies have used dental caries as the endpoint measurement. This study was designed as a randomized double-blinded clinical long-term trial on the effects of *L. paracasei* SD1, a human oral-origin strain, on both MS level and dental caries. The number of children investigated in this study is given in the flow chart of Figure 1. The baseline characteristics of the children in both groups who participated in the study are presented in Table 1. There were no statistically significant differences between the groups in age, pH of saliva and caries status (DMFT) at the baseline (*P* > 0.05). The sex in both groups was significantly different (*P* < 0.05); there were more females in the probiotic group than in the control group. The dropout rate from the baseline was low, and compliance with the study protocol during the intervention was satisfactory based on the information from the logbook.

**Table 1:** General characteristics of children.

Characteristics	Control	Probiotic
Age (years)	13.13 ± 0.71	13.25 ± 0.73
Sex:		
male	41	23
female	22	36
DMFT at baseline	0.91 ± 1.40	0.86 ± 1.12
pH of saliva:		
T0	7.47 ± 0.29	7.50 ± 0.30
T3	7.18 ± 0.42	7.38 ± 0.49
T6	7.60 ± 0.60	7.78 ± 0.36
T9	7.70 ± 0.41	7.57 ± 0.49
T12	7.32 ± 0.47	7.30 ± 0.56

**Figure 2:**
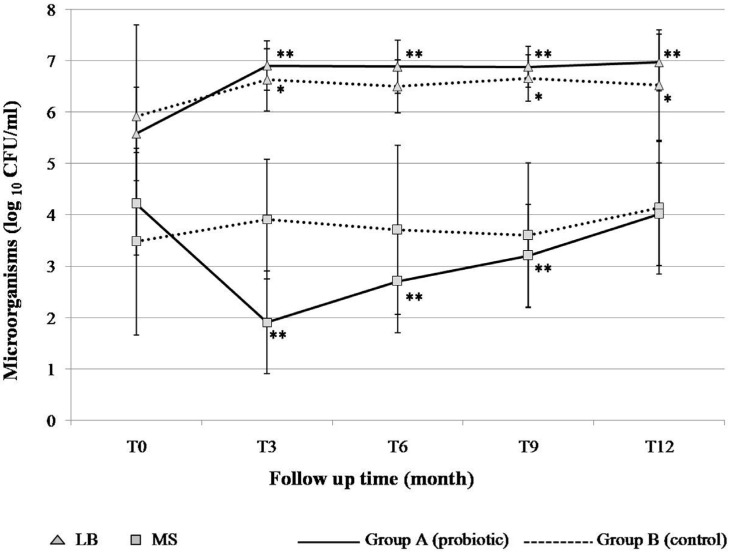
The number of salivary mutans streptococci (MS) and lactobacilli (LB) at the baseline (T0) and during the 12 months of study (T3–T12). The parameters were evaluated by the Wilcoxon signed-rank test: * Significance (*P* < 0.05), ** Significance (*P* < 0.001) difference *versus* baseline.

In previous studies, the follow-up samplings were generally conducted at the termination of probiotic intervention, and any information on the post intervention re-growth of the suppressed target bacteria afterwards was minimal. This study monitored the MS levels more frequently during the intervention and follow-up time. It was hypothesized that long-term exposure to probiotics could prevent or delay MS colonizing, and accordingly prevent dental caries. Thus, evaluation of the study was based on MS colonization and the increment of dental caries between the children receiving probiotic milk and control milk. The levels of salivary MS and lactobacilli at baseline and at the end of the study are demonstrated in Figure 2. At baseline, the mean counts (log_10_ CFU/mL ± SD) of MS in the probiotic and control groups were 4.21 ± 1.01 and 3.48 ± 1.82, respectively, which was not a statistically significant difference (*P* > 0.05). Significant differences of MS levels between the two groups were found at T3 (*P <* 0.001) and T6 (*P* = 0.001). The MS levels did not change significantly among children taking the control milk throughout the study, except that it significantly increased during T9–T12 (*P* = 0.011). The number of MS in the saliva of children in the probiotic group was found to have significantly decreased during and three months after receiving the probiotic milk (T3–T9) compared to the baseline (*P* < 0.001). At the end of the study (T12), the MS level was not significantly different compared to the baseline. It was assumed that the presence of *L. paracasei* SD1 resulted in having an inhibiting effect on MS. However, it should be noted that probiotic action involves several mechanisms including production of antimicrobial substances, competition with pathogens by preventing cellular adhesion and invasion, and modulation of local and systemic immune functions [19]. We have also found a significant increase of salivary innate immune among children receiving the probiotic milk at T3 (published separately). So far, there is no clear explanation for the observation why there was a rapid decrease of MS at T3, it might be the result of several mechanisms of probiotic action in combination. This requires further elucidation.

At baseline, the mean counts of lactobacilli in the probiotic and control groups were 5.57 ± 0.91 and 5.92 ± 1.77, respectively, being significantly lower in the probiotic group than in the control group (*P*<0.001). The significant differences of lactobacilli counts between both groups were found at T3 (*P* = 0.027), T6 (*P* = 0.003), T9 (*P* = 0.043) and T12 (*P* = 0.027). During the follow-up period, the level of lactobacilli increased significantly in both groups compared to the baseline; however, it increased more in the probiotic group than in the control group.

**Figure 3:**
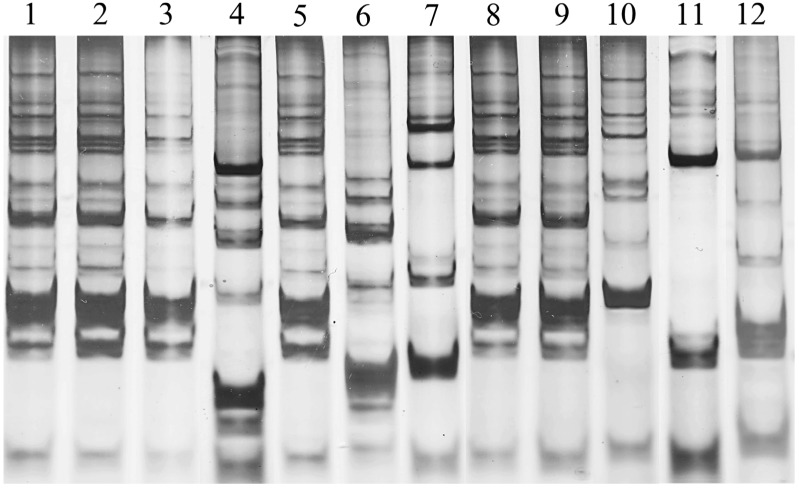
DNA fingerprint profiles of *L. paracasei* SD1 contained in milk powder (lane 1) and 11 clinical lactobacilli strains (lanes 2–12) isolated from saliva of four individual children who received milk contained *L. paracasei* SD1 at T9 of study: lanes 2–4, lanes 5–7, lanes 8–10, and lanes 11–12 isolates from child 1, 2, 3 and 4, respectively. The child 1, 2 and 3 had the isolates in lanes 2, 3, 5, 8 and 9, which showing the DNA fingerprint profiles similar to *L. paracasei* SD1. *L. paracasei* SD1 could not be detected in the child 4.

The finding of the increase of lactobacilli counts raised the questions of whether those were the probiotic bacteria and whether the probiotic bacteria could permanently colonize in the mouth or not. In this study, overall lactobacilli counts were monitored, with a focus on tracing for *L.* *paracasei* SD1. Thus, the DNA fingerprinting pattern was analyzed for *L.* *paracasei* SD1 persistence among the lactobacilli strains isolated from saliva of children in both groups. The method has been previously proven to be useful for genotyping of *Lactobacillus* strains and unique DNA fingerprinting pattern was found in each individual strain (10). The presence of *L. paracasei* SD1 was found only in the children after receiving the milk contained probiotic strain (group A). At baseline, *L. paracasei* SD1 was not detectable in either the control or test group. During the study, the finding of *L. paracasei* SD1 could be detected as 85%, 80%, 60% and 0% at T3, T6, T9 and T12, respectively, in the children who were received probiotic milk (examples shown in Figure 3). *L. paracasei* SD1 was not found in the control group throughout the study. The result was similar to the previous study (10), *L. paracasei* SD1 could be found (75% of volunteers who received probiotic milk) up to four weeks following cessation of dosing. However, the present study showed that *L. paracasei* SD1 could not be detected at T12 (six months following cessation of dosing).

Some studies showed that different strains of probiotic strains act as a transient colonizer in the oral cavity, and studies on plaque and saliva have shown that ingested probiotic bacteria were recoverable up to one to two weeks after termination of intake [7,8,9]. However, the prolonged time for colonization of *L. paracasei* SD1, of at least three months (T6–T9) after cessation of dosing, in the mouth of the children taking the probiotic milk was noted in the present study. The colonization of *L. paracasei* SD1 seemed to last longer than the other studies [7,8,9]. This may relate to its origin from the human mouth, where the environment is more suitable for the strain to survive. Another possibility is that prolonged exposure to the probiotic strains may be needed. Our previous study evaluated the short-term (four weeks) consumption of milk containing *L. paracasei* SD1, and demonstrated that the strain could be detected for a further four weeks afterwards. A short contact time of an extrinsically administered probiotic to the oral cavity may be a limiting factor, since the already established microbial ecology may prevent any introduced probiotics from colonizing and becoming part of the commensal oral biofilm.

Any residual effect after a long-term taking of products containing probiotics has always been an issue of interest. The establishment of probiotic strains in the oral cavity could promote caries development due to their acidogenic and aciduric properties. However, *L. paracasei* strains have been shown being less acid-producers compared to strong acid producers e.g. *Lactobacillus salivarius* [20]. In this study, lactobacilli level significantly increased in both groups, but the increase was found much more in the probiotic than in the control group. All participants were advised not to take food or drink containing probiotic (lactobacilli) during the study. However, an increase of lactobacilli levels in the control group might be derived from an unexpected source, e.g. pickled vegetables and fruits, which is commonly ingested among Thai people. No similar DNA fingerprint profiles as *L. paracasei* SD1 were found among children who received the control milk; this indicated that the recovered lactobacilli was derived from other sources. It was noted that there were no negative side effects following the probiotic intervention. Although fluctuations in saliva pH were observed, they were within the neutral range. Throughout the study, the salivary pH was found closely the same from the baseline to the end of the study in both groups (Table 1).

There have been only a few reports of randomized controlled clinical trials using caries as the outcome, especially regarding clinical evaluation of probiotic bacteria that originated from the mouth. Most strains that have been isolated and developed for gastrointestinal health have been adopted for use in dental research. The six-month intervention followed by six months observation in this study may still be too short for monitoring caries progression. Fluoride is commonly used in Thailand, which may be another factor delaying caries progression. However, our results revealed that *L.* *paracasei* SD1 showed a beneficial effect on dental caries; there was less dental caries among the children in the probiotic group at the end of study (Table 2). The mean of decay teeth (DT) in the probiotic group was lower than in the control group, although there was no statistically significant difference (Table 2). When the caries increment (∆ DT) and caries risk were taken for analysis, a significant increase in the caries increment was observed among children in the control group with high caries risk compared to the probiotic group (Table 2). In addition, it revealed that the long-term intake of milk containing *L. paracasei* SD1 reduced the risk of caries significantly (OR = 4.55, *P* = 0.019) in children with high caries risk (Table 3). Among children with low caries risk, it seemed that the probiotic group experienced a greater increase in caries (≥1) than the control group, however, it was not statistically significant (OR = 0.45, *P* = 0.342) (Table 3). The definition of caries risk here is based on either dental caries alone or combined with microbiological examination. This is in agreement with another study of a caries trial reporting the effect of probiotics in reduction of caries risk and caries incidence [12]. Theoretically, a reduction of MS over time would reduce caries risk, at least of new lesions. The clinical significance of our findings supported that a long-term exposure to milk containing *L. paracasei* SD1 could prevent or delay MS colonization resulting in reduction of caries risk.

**Table 2:** Mean decay teeth (DT) ± SD at T0 and mean caries increments (∆ DT) ± SD at T12 in the control and probiotic groups.

Dental caries	Control group	Probiotic group
Total: (n = 110)		
DT at T0	0.61 ± 1.10	0.68 ± 0.98
∆ DT at T12	0.57 ± 0.95	0.30 ± 0.57
Low caries risk: (n = 56)		
DT at T0	0.0	0.0
∆ DT at T12	0.30 ± 0.79	0.35 ± 0.56
High caries risk: (n = 54)		
DT at T0	1.43 ± 1.31	1.26 ± 1.03
∆ DT at T12*	0.91 ± 1.04	0.26 ± 0.57

Note: * Significance (p = 0.007), the parameters were evaluated by the Mann-Whitney U test.

**Table 3:** Number of children with caries increments of the control and probiotic groups with low and high caries risk.

Dental caries	Number of children (%) with			
	Low caries risk (n = 56)		High caries risk (n = 54)*	
	Control	Probiotic	Control	Probiotic
No caries increase	25 (83.3)	18 (69.2)	11 (47.8)	25(80.6)
Caries increase ≥ 1	5 (16.7)	8 (30.8)	12 (52.2)	6 (19.4)
Total	30 (100)	26 (100)	23 (100)	31 (100)
OR	0.45		4.55	

Note: * Significance (p = 0.019), the parameters were evaluated by the Chi-Square test.

It has been accepted that the effects of probiotic strains are strain specific, not all the *Lactobacillus* are probiotics that possess the ability to confer health benefits for the host [21]. Thus, exploring the desirable properties of certain strains is needed. Our previous study revealed that *L. paracasei* SD1 could produce paracasin SD1, a specific antimicrobial protein, against various oral pathogens including cariogenic bacteria [22]. It may support the advantages of this strain in respect to its potential use as a bacterial replacement, which is a means of combatting infections by the administration of non-pathogenic bacteria to displace pathogenic microorganisms.

## 3. Experimental Section

### 3.1. Subjects

To be considered for invitation, subjects had to have good oral health with caries ≤3 teeth, absence of untreated active carious lesions, absence of either gingivitis or periodontal disease, be a non-smoker, and have daily tooth-brushing habits using fluoride-containing toothpaste. The exclusion criteria were (i) habitual consumption of probiotics or xylitol; (ii) systemic antibiotic medication taken within six weeks; (iii) allergy to cows’ milk, lactose intolerance, severe food allergy; and (iv) systemic or severe chronic diseases.

The sample size calculation was based on our previous study [11]; it was calculated that there would be an estimated 80% power at the 0.05 level of significance, using two-sided testing, to detect a mean difference on the logarithm scale of 0.55 for MS count assuming a standard deviation of 0.63. Twenty participants per group were needed. After adjustment to evaluate the result of the caries increment and for 20% dropouts, a total sample size of 120 was judged as necessary for this study.

The project was thoroughly explained to the children and their parents in a meeting organized at the school. From a total of 246 children, 202 were eligible but only 122 volunteered after informed consent was given by their parents. The study group comprised 122 children (58 females, 64 males), 12–14 years of age (mean: 13.19 ± 0.72 years). A flow chart of the study is outlined in Figure 1. All subjects were asked to immediately report any adverse side effects and to fill in the questionnaire form after six months of milk consumption.

### 3.2. Study Design

The prospective investigation was a follow-up of a double-blinded, randomized placebo-controlled trial in two parallel groups, with a study period of 12 months. The study was approved by the Faculty of Dentistry Ethics Committee at the Prince of Songkla University, Thailand. Children were randomized to the study or control group by means of drawing lots, and they were coded as A or B. The code was kept by an independent monitor. This code was not unveiled until all data had been analyzed. Neither the researchers nor clinicians and health care personnel knew whether the children received control or intervention milk during the course of the study.

### 3.3. Intervention

Children in the probiotic group drank 5 g of reconstituted milk powder with probiotic *L. paracasei* SD1 in 50 mL water once daily, whereas children in the control group drank 5 g of reconstituted milk powder without probiotic bacteria in 50 mL water once daily for six months. The probiotic milk powder contained *L. paracasei* SD1 10^7^ CFU/mg and was prepared according to Teanpaisan *et al.* [23]. The test and the control milks were delivered in plastic bags marked “A” or “B”, and all children were asked to drink under observation everyday by the health care staff. On the holidays or weekends, children were asked to drink their milk at home and to return the plastic bags.

The compliance was checked by the health care staff who filled in a logbook everyday with information on attendance of children and whether or not the children had been drinking the milk.

### 3.4. Oral Examination

Oral examinations were performed at baseline and at the end of the study after 12 months by three dentists (Supatcharin Piwat, Sukanya Tianviwat, and Thanyanan Kampoo). Before the start of the study, the three examination teams were calibrated against each other and inter-examiner reliability tests were carried out before the baseline and before the re-examination.

Dental caries status was recorded according to WHO criteria [24]. The caries increment (∆ DT) was calculated for each participating individual as the difference between the baseline score and the 12-month score.

Caries risk was determined on the basis of combined clinical and microbiological results according to the modification of Nase *et al.* [12], which classified the children into “high risk” and “low risk”. “High risk” was recorded if the child had either a DT score > 0 and/or MS count ≥10^5^ cfu/mL. If there was no caries (DT score = 0) and the MS count < 10^5^ cfu/mL, the child was recorded as “low risk”.

### 3.5. Microbial Evaluation

Unstimulated saliva samples were collected at baseline (T0), 3 (T3), 6 (T6), 9 (T9) and 12 (T12) months. Each sample was made in a 10-fold serial dilution and cultured on a Mitis Salivarius Bacitracin agar or a de Man, Rogosa and Sharpe (MRS) agar being the selective media for MS and lactobacilli, respectively. After 48 h of incubation in anaerobic condition at 37 °C, the number of MS and lactobacilli were counted as colony forming units (CFU)/mL.

Five to ten colonies of lactobacilli on MRS plates were collected, purified and kept at −80 °C for tracing of *L. paracasei* SD1 using AP-PCR fingerprints [10].

### 3.6. Analysis of Data

All numerical data were presented as means and standard deviations. The general characteristics of the children (gender, age, pH of saliva and caries status-DMFT) between control and probiotic groups were analyzed using the chi-square test for categorized/dichotomized variables and the Mann-Whitney U test for interval variables. The number of colony counts of MS and lactobacilli were presented as log_10_ CFU/mL. The changes in bacterial counts and salivary pH from baseline to the intervention period were analyzed using Wilcoxon Signed rank test. The number of colony counts of MS, and lactobacilli between the two groups were analyzed using the Mann-Whitney U test. The difference of caries increment between the two groups was analyzed by the Mann-Whitney U test. The chi-square test was used to compare the percentages of children with caries increment between the study groups. The software package used was the Statistical Package for Social Sciences (SPSS Inc., Chicago, IL, USA), and the differences were considered significant when *P* < 0.05.

## 4. Conclusions

In conclusion, the long-term daily consumption of milk powder containing *L. paracasei* SD1 has resulted in the reduction of both numbers of MS in saliva and caries risk. Long-term exposure seems to be an essential factor for prolonged colonization of the probiotic strain. Therefore, prolonged administration or the repeated application of probiotics may be needed to maintain the desired probiotic effect to have benefit to oral health.
